# Overexpression of Phosphoserine Aminotransferase (*PSAT*)-Enhanced Cadmium Resistance and Accumulation in Duckweed (*Lemna turionifera* 5511)

**DOI:** 10.3390/plants13050627

**Published:** 2024-02-25

**Authors:** Xu Ma, Yumeng Jiang, Ziyang Qu, Yunwen Yang, Wenqiao Wang, Yuman He, Yiqi Yu, Ximeng Luo, Yuanyuan Liu, Wenqian Han, Qiqi Di, Lin Yang, Yong Wang

**Affiliations:** 1 Tianjin Key Laboratory of Animal and Plant Resistance, College of Life Sciences, Tianjin Normal University, Tianjin 300387, China; 2 College of Life Science, Nankai University, Tianjin 300071, China

**Keywords:** duckweed, phosphoserine aminotransferase, glutamate, cadmium, accumulation

## Abstract

Cadmium (Cd) hampers plant growth and harms photosynthesis. Glutamate (Glu) responds to Cd stress and activates the Ca^2+^ signaling pathway in duckweed, emphasizing Glu’s significant role in Cd stress. In this study, we overexpressed phosphoserine aminotransferase (*PSAT*), a crucial enzyme in Glu metabolism, in duckweed. We investigated the response of *PSAT*-transgenic duckweed to Cd stress, including growth, Glu metabolism, photosynthesis, antioxidant enzyme activity, Cd^2+^ flux, and gene expression. Remarkably, under Cd stress, *PSAT*-transgenic duckweed prevented root abscission, upregulated the expression of photosynthesis ability, and increased Chl a, Chl b, and Chl a + b levels by 13.9%, 7%, and 12.6%, respectively. Antioxidant enzyme activity (CAT and SOD) also improved under Cd stress, reducing cell membrane damage in *PSAT*-transgenic duckweeds. Transcriptomic analysis revealed an upregulation of Glu metabolism-related enzymes in *PSAT*-transgenic duckweed under Cd stress. Moreover, metabolomic analysis showed a 68.4% increase in Glu content in *PSAT* duckweed exposed to Cd. This study sheds novel insights into the role of *PSAT* in enhancing plant resistance to Cd stress, establishing a theoretical basis for the impact of Glu metabolism on heavy metal tolerance in plants.

## 1. Introduction

In plants, various strategies are employed to immobilize and accumulate Cd. Firstly, phytochelatins (PCs), with a [(γ-Glu-Cys)n]-Gly structure, can form PC-Cd complexes, effectively immobilizing Cd. Secondly, during Cd stress, the levels of proline (Pro), histidine (His), and glutathione (GSH) have been observed to increase [1]. Thirdly, previous studies have shown an increase in Glu content, highlighting its importance as a signal during Cd stress responses in duckweed [2,3]. Glu responds to stress conditions, such as wounds, pathogen infections, cold, salt, and heat [4]. These studies have shown that Glu functions as a metabolite, but also as a signal molecule. Interestingly, glutamate (Glu) has been reported to form Glu-Cd complexes to mitigate Cd toxicity [5]. Glu serves as a precursor for stress-responsive amino acids, enhancing heavy metal tolerance in plants [6]. Glutamate synthesis plays a pivotal role in determining Cd accumulation in rice grains [7]. However, the functions of enzymes involved in Glu metabolism during Cd accumulation remain unclear.

Glu can be synthesized by four different enzymes. Firstly, Glu can be produced by glutamate dehydrogenase (GDH, E.C. 1.4.1.3.), which regulates Glu metabolism [8]. Secondly, Glu can be converted from glutamine (Gln) through the action of glutamate synthase (GOGAT, EC:1.4.1.13.) [9]. Thirdly, in the photorespiration pathway, glutamate: glyoxylate aminotransferase (GGAT, EC 2.6.1.4) catalyzes the reaction between glutamate and glyoxylate to yield 2-oxoglutarate and glycine. Glu content was decreased in Arabidopsis with GGAT1 overexpression and increased in the knockout line (ggat1-1), indicating that Glu serves as an amine donor for the GGAT reaction [10]. Moreover, 3-phosphoserine aminotransferase (*PSAT*, EC:2.6.1.52.) plays a crucial role in glutamate metabolism by facilitating the interconversion of Glu and 3-phosphohydroxypyruvate (3-PHP) into 2-oxoglutaric acid and 3-phosphoserine [11]. *PSAT* contributes to serine biosynthesis in plants and other organisms [12,13]. Serine serves as a precursor for tryptophan, glycine, and cysteine, which are essential for nucleic acid and protein biosynthesis [14]. Furthermore, *PSAT* has been identified as a driver of starch accumulation in duckweed under nitrogen-starved conditions [15]. In our previous study, significant upregulation in the expression of both *PSAT* and GDH was observed during Cd stress [2]. Investigating the impact of *PSAT* overexpression in response to Glu signaling under Cd stress holds promising implications for enhancing Cd tolerance.

Glutamate (Glu) plays multiple roles beyond being a simple metabolite, displaying diverse functions. Firstly, Glu can initiate a response similar to elicitors in plants, mediated by glutamate receptor-like proteins (GLRs). GLRs include cation-permeable ion channels, such as calcium (Ca^2+^). Acting as a defensive signal, Glu triggers long-distance Ca^2+^-based responses in plants during wound defense [16]. Secondly, Glu acts as a precursor for gamma-aminobutyric acid (GABA) and ornithine (Orn). Ornithine, in turn, serves as a precursor for various polyamines vital for plant development. GABA, known as an inhibitory neurotransmitter in animals, interacts with GABA (ionotropic) receptors, contributing to stress responses [2]. Maintaining Glu balance is crucial for cellular physiology under stress, as low concentrations respond to signaling stimulation [16], while high concentrations may lead to cell damage [17]. Therefore, the possibility of enhancing cellular tolerance under stress through the overexpression of relevant metabolic enzymes in regulating Glu homeostasis holds great promise. Investigating Glu’s function, particularly when modulated by enzyme regulation during Cd stress, carries significant importance in enhancing Cd tolerance.

We hypothesized that *PSAT* plays a crucial role in regulating Glu content and its response during Cd stress. This study aims to accomplish the following objectives: (i) to overexpress *PSAT* in duckweed to investigate Cd tolerance and Cd accumulation in transgenic duckweed; (ii) to analyze metabolic pathways and amino acid changes in transgenic duckweed under Cd stress; (iii) and to explore the regulation of genes associated with the Glu metabolism pathway in *PSAT*-expressing duckweed under Cd stress.

## 2. Results

### 2.1. Construction and Identification of PSAT Transgenic Duckweed

**Figure 1:**
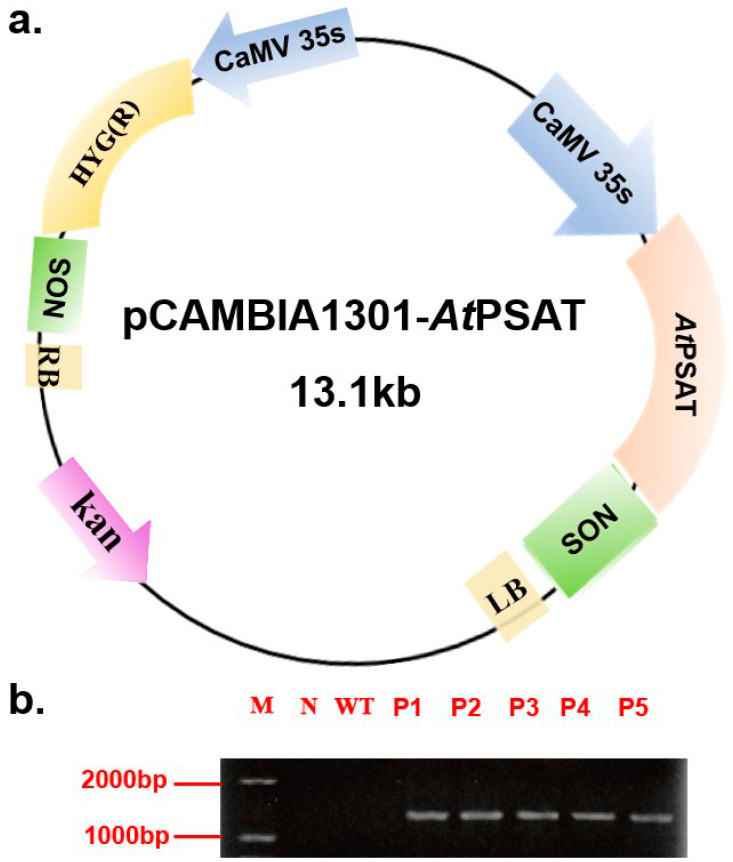
Construction of the pCAMBIA 1301-*AtPSAT* recombinant vector and the identification of transgenic duckweed. (**a**) Schematic representation of the pCAMBIA 1301-*AtPSAT* recombinant vector. Kan.: Kanamycin resistance. HYG: hygromycin resistance. (**b**) Specific PCR amplification for *AtPSAT* identification. N: Negative control, where water served as the template for PCR; WT: wild type; P1-5: *AtPSAT*1-5.

The recombinant vector was constructed and subsequently introduced into duckweed callus via *Agrobacterium tumefaciens* GV3101. Hygromycin was used for the selection of transformed plants during leaf regeneration. Following this, genomic DNA was extracted from the plants and subjected to specific PCR amplification targeting the *PSAT* gene (AGI number AT4G35630) for identification purposes. Notably, *PSAT* was not amplified in the negative control or the wild-type (WT) plants. These results confirmed the successful transformation of all five transgenic strains (Figure 1b). For subsequent experiments, the *PSAT*-4 transgenic strain was chosen.

### 2.2. Over-Expression of PSAT1 Improved Cd Tolerance, Photosynthetic Relative Genes Expression, and Chlorophyll Content during Cd Stress

**Figure 2:**
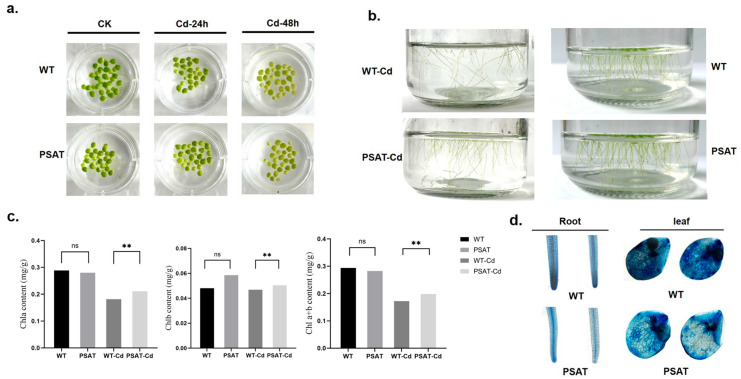
Phenotypic observations, chlorophyll content, and membrane damage assessed by Evans Blue staining in PSAT and WT duckweed. (**a**) Leaf yellowing in PSAT and WT after exposure to 50 μM Cd for 24 and 48 h. (**b**) Root abscission in PSAT and WT after 24 h of treatment with 50 μM Cd. (**c**) Chlorophyll a, chlorophyll b, and total chlorophyll a + b content in WT and PSAT after 48 h of exposure to 50 μM Cd. Significant differences were analyzed using independent samples *t*-test and are indicated by asterisks (** *p* < 0.01), ns: not significant. (**d**) Evans Blue staining of roots and leaves of WT and PSAT following a 48-h treatment with 50 μM Cd.

After 24 h of exposure to Cd treatment, there was no significant difference in leaf yellowing between the *PSAT* and WT duckweed. However, following 48 h of Cd stress, the leaves of the WT duckweed exhibited noticeable yellowing (Figure 2a). Importantly, the *PSAT*-transgenic duckweed showed a significantly lower root abscission rate (15.69%) compared to the WT (29.79%). The root abscission rate was calculated using the following formula: (Number of roots abscised/Total number of roots × 100%) (Figure 2b). Additionally, Evans blue staining results indicated that under Cd stress, *PSAT*-transgenic duckweed exhibited lighter staining compared to the WT, suggesting enhanced protection of cell membrane integrity (Figure 2d).

Chlorophyll plays a vital role in plant photosynthesis. The levels of chlorophyll a (Chla), chlorophyll b (Chlb), and total chlorophyll a + b (Chla + b) were measured in WT and *PSAT* duckweed with or without Cd stress at 48 h. In the absence of Cd treatment, there was no significant difference in the chlorophyll content between *PSAT* and WT. However, under Cd stress, *PSAT* plants displayed a significant increase in Chla, Chlb, and Chla + b levels, with increments of 13.9%, 7% and 12.6%, respectively (Figure 2c). Furthermore, transcriptome analysis revealed that *PSAT* led to a notable upregulation in the expression of genes associated with Photosystem II, Photosystem I, Photosynthetic electron transport, and F-type ATPase compared to the WT during Cd stress (Table 1).

### 2.3. An Enhanced Antioxidant Capacity in PSAT Duckweed under Cd Stress

**Figure 3:**
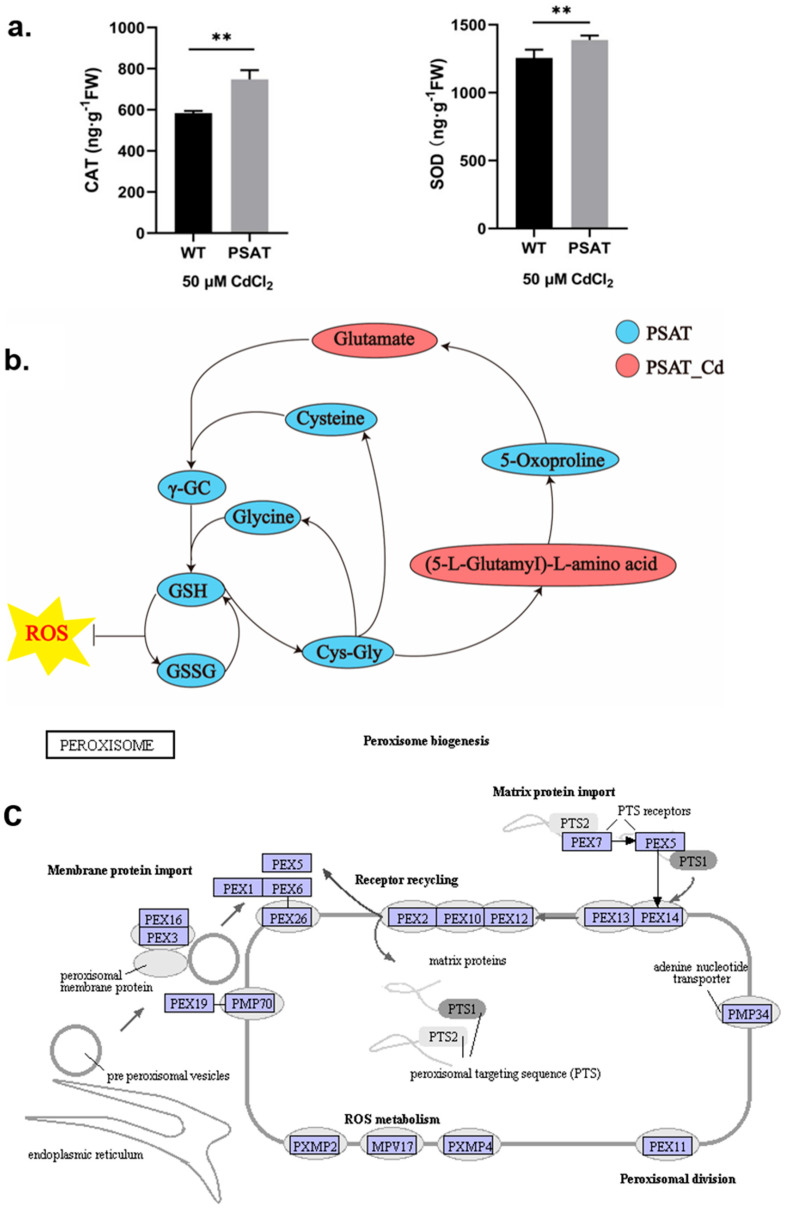
Study of antioxidant capacity, GSH content, and peroxisome gene expression in *PSAT*− transgenic duckweed during Cd stress. (**a**) Levels of CAT and SOD in WT and *PSAT* treated with 50 μM Cd for 48 h. Significant differences were analyzed using independent samples *t*-test and are indicated by asterisks (** *p* < 0.01). (**b**) Key metabolites related to GSH mapped onto the KEGG metabolite pathway map. The colors represent metabolites which were significantly elevated in the group (green: *PSAT*; red: *PSAT*_Cd). (**c**) Changes in peroxisome gene expression under Cd stress. The red box indicates upregulation. Different letters within the same column indicate significant differences between treatments at *p* ≤ 0.05 using SPSS.

During Cd stress, *PSAT* demonstrated significantly higher levels of CAT and SOD content in comparison to the WT (Figure 3a). Notably, *PSAT* exhibited the highest glutathione (GSH) content (Figure 3b and Figure S1). GSH is the primary sulfhydryl group (−SH) and the most abundant low-molecular-weight polypeptide in cells. It plays a crucial role in activating the biological REDOX system and sulfhydrolases, effectively eliminating oxygen free radicals (ROS) from tissues. This result highlights the enhanced ability of *PSAT*-transgenic duckweed to remove ROS. In Figure 3c, the gene expression of mitochondrial inner membrane protein MPV17 and peroxisomal membrane protein PMP34 showed significant upregulation in *PSAT*-transgenic duckweed under Cd stress.

### 2.4. The Expression of Genes Related to Glu Metabolism Pathway in PSAT Duckweed under Cd Stress

**Figure 4:**
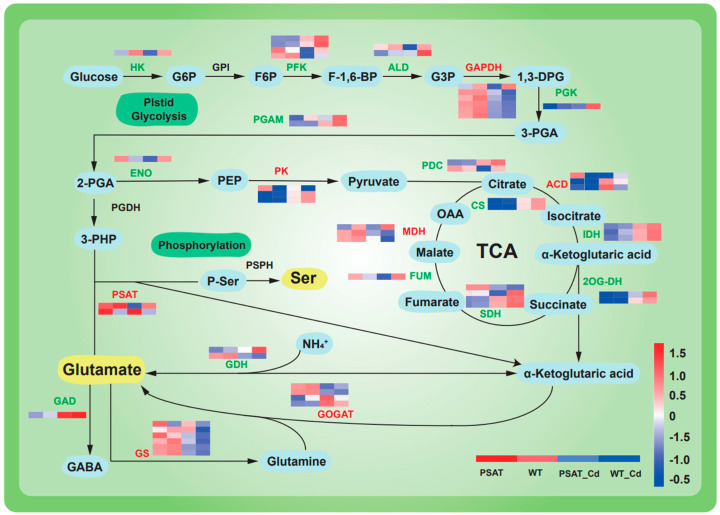
Heatmap depicting gene expression patterns associated with glycolysis, the tricarboxylic acid (TCA) cycle, and glutamic acid and serine metabolic pathways in *PSAT*−transgenic and WT duckweed under different conditions, with or without Cd stress. The color scale ranges from red (high expression) to blue (low expression), with changes indicated by log2 Fold values. Data reprsent means ± SD, *n* = 3.

The expression of genes linked to glycolysis and the tricarboxylic acid (TCA) cycle was upregulated under Cd stress in both *PSAT*-transgenic and WT duckweed when compared to conditions without Cd stress (Figure 4). Moreover, in the *PSAT*_Cd group, genes involved in the glutamate metabolism pathway exhibited upregulation compared to the WT_Cd group. This included glutamate synthase (GOGAT), with a 0.31 log2 Fold change; glutamine synthetase (GS), with a 1.51 log2 Fold change; and phosphoserine aminotransferase (*PSAT*), with a 1.90 log2 Fold change, among others (Table S1).

### 2.5. The Changes in Metabolism Analysis of Pathway Enrichment and Amino Acid Content in PSAT Duckweed during Cd Treatment

**Figure 5:**
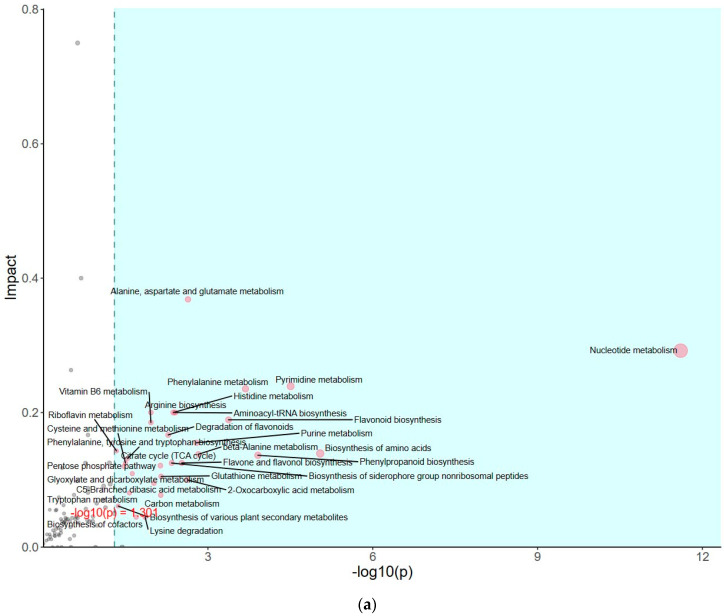

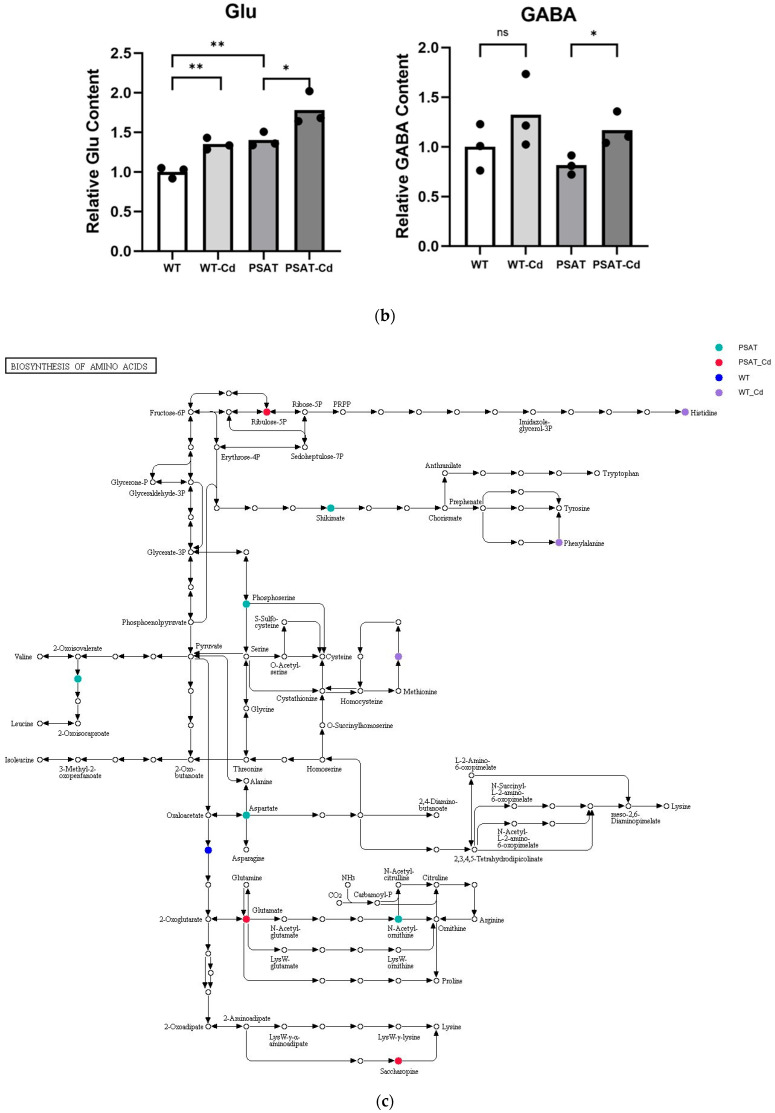
Study of metabolite contents in *PSAT* and WT duckweed under various conditions. (**a**) Scatterplot illustrating the significance of metabolite differences. (**b**) Relative contents of Glu and GABA in WT/*PSAT* under Cd stress. Relative content of Glu and GABA in *PSAT* duckweed with or without Cd stress. Significant differences were analyzed using independent samples *t*-test and are indicated by asterisks (* *p* < 0.05, ** *p* < 0.01), ns: not significant. (**c**) Enrichment analysis of amino acids in the metabolomic analysis. The color indicates metabolites with significantly higher levels in the group (green: *PSAT*; red: *PSAT*_Cd).

The impact of pathways on metabolite contents was investigated between *PSAT* and WT duckweed under Cd stress across various metabolic processes (Figure 5a). Several of these metabolic processes are closely linked to plant responses to cadmium stress, including those involving glutamate, glutathione, and the tricarboxylic acid (TCA) cycle. As shown in Figure 5b and Table S2, metabolomics analysis revealed that *PSAT* exhibited the highest Glu content under Cd stress. Furthermore, the overexpression of *PSAT* led to an increase in Glu content, which was 1.4 times higher than that in the WT. Interestingly, the Glu content increased during Cd treatment, with the Glu content in both WT and *PSAT* duckweed under Cd stress (WT-Cd group; *PSAT*-Cd group) being 1.35 and 1.78 times higher than that in the WT. Amino acid synthesis was also analyzed (Figure 5b; Table S2). Glu metabolism is closely associated with the cycling of glutathione and ascorbic acid, both of which play essential roles in antioxidant processes. This result aligns with the role of the Glu signal in responding to Cd stress. Notably, *PSAT* duckweed exhibited enriched content in shikimate, phosphoserine, 2-Oxoisovalerate, aspartate, and N-Acetylcitrulline. Additionally, *PSAT* under Cd stress increased the levels of ribulose, which is crucial for photosynthesis.

### 2.6. Cd Absorption Was Improved in PSAT Duckweed

**Figure 6:**
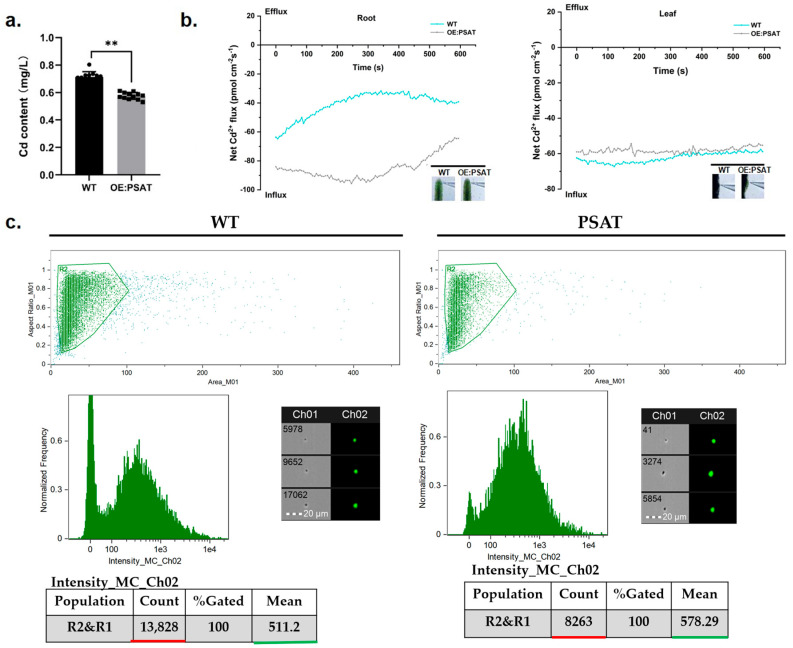
Study of Cd content in water, Cd^2+^ flux, and Cd uptake in *PSAT* and WT duckweed. (**a**) Cd content in the water cultured with duckweed (WT/*PSAT*). Significant differences were analyzed using independent samples *t*-test and are indicated by asterisks (** *p* < 0.01). (**b**) Net Cd^2+^ flux in the roots and leaves of wild type and *PSAT,* measured using NMT after treatment with 50 μM Cd for 30 min. (**c**) Cd fluorescence intensity in protoplasts from roots, analyzed using a flow sight system at 488 nm. Both WT and *PSAT*−transgenic duckweed were soaked in 50 μM Cd for 24 h. Leadmium^TM^ Green AM was used to stain their protoplasts. The bright field is Ch 01, while the 488 nm excitation light is Ch 02, with a scale bar of 20 µm. The protoplasts, framed in green, were selected to analyze fluorescence intensity.

To evaluate the Cd adsorption capacity of *PSAT*-transgenic duckweed, the Cd content in the treatment solution was quantified. After both WT and *PSAT* were cultured in 50 μM Cd for 24 h, the Cd content in the WT treatment solution was 0.713 mg/L, while *PSAT* reduced it to 0.576 mg/L (Figure 6a). Clearly, *PSAT* demonstrated a stronger ability to remove Cd from the water. Real-time Cd fluxes in the roots and leaves of WT and *PSAT* were measured in a Cd solution using the NMT assay. In both the roots and leaves, Cd^2+^ influx was observed (Figure 6b). There was no significant difference in Cd^2+^ influx between WT and *PSAT* in the leaves. However, the absorption of Cd by the roots of *PSAT* was significantly higher than that of WT, peaking at 250–300 s before declining. Furthermore, Cd fluorescence values in the roots of WT and *PSAT* were detected after Cd staining using a flow sight system (Figure 6c). The results revealed that the fluorescence intensity of Cd in the roots of *PSAT* (mean = 578.29) was significantly higher than that of WT (mean = 511.2), indicating a 68.4% increase in Cd absorption.

## 3. Discussion

### 3.1. Overexpression of PSAT Improved Cd Resistance by Protecting Root and Enhancing Photosynthesis

Roots of various plant species are susceptible to the detrimental effects of Cd stress, often resulting in symptoms such as browning, reduced root length, and increased root diameter [17,18,19]. In our previous research, we observed that under conditions of salt and Cd stress, duckweed roots tended to undergo fracturing. This phenomenon could potentially act as a barrier, preventing the translocation of salt or cadmium to other leaf regions and thereby mitigating extensive damage [19]. In the present study, root breakage was significantly reduced in the *PSAT*-overexpressing duckweed compared to the WT (Figure 2b). Furthermore, external stress led to a reduction in the number of leaf clusters in duckweed. Interestingly, although there was no significant difference in leaf yellowing between *PSAT* and WT, *PSAT* exhibited a higher count of leaf clusters (Figure 2b). These results indicate an enhanced resistance to Cd stress in *PSAT*-overexpressing duckweed.

The chlorophyll content and photosynthetic system capacity serve as physiological sensitivity indicators in plants exposed to Cd stress [20]. Cd stress typically results in a decrease in chlorophyll content due to the promotion of degradation or the inhibition of synthesis [21]. Previous studies have reported the impact of Cd stress on chlorophyll concentration in star clusters (*Pentas lanceolata*) [18]. Additionally, Cd disrupts photosynthetic processes by impeding chlorophyll synthesis and its stable association with proteins, particularly the light-harvesting complex, photosystem I (PSI), and photosystem II (PSII) [22]. In this study, the overexpression of *PSAT* increased the levels of chlorophyll a, b, and total chlorophyll in duckweed under cadmium stress (Figure 2c). At the transcriptome level, the gene expression levels of PSII, PSI, photosynthetic electron transport, and F-type ATPase were enhanced (Table 1). Taken together, these findings suggest that *PSAT* upregulates the expression of genes related to PSII and PSI, and helps to maintain chlorophyll integrity, contributing to improved photosynthesis and Cd resistance.

### 3.2. PSAT Promoted Glutamate Metabolism and Cd Accumulation

Glutamate (Glu) functions as a signaling molecule in response to external damage, playing a pivotal role, as highlighted in recent reports that elucidate its positive influence on plant stress regulation [2,5,7,16]. *PSAT* serves as a crucial key enzyme in Glu synthesis. 3-PHP binds to Glu and is catalyzed by *PSAT* to produce phosphoserine (P-Ser) and α-ketoglutaric acid. In the context of oncology, *PSAT* plays a vital role in the development of estrogen receptor (ER)-negative breast cancer [23]. Silencing *PSAT* inhibits the movement and migration of triple-negative breast cancer (TNBC) and reduces the number of tumors [24]. Additionally, *PSAT*1 stimulates colorectal cancer cell proliferation and modulates chemotherapy sensitivity both in vitro and in vivo [25]. In plants, the structure of *PSAT*1 has been analyzed in *Arabidopsis thaliana* [11]. This research suggests that the movement of the gate-keeping loop (residues 391–401) is a key element in regulating the catalytic activity of *PSAT* when 3-PHP binds to P-Ser. Overexpression of *PSAT*1 in duckweed (*Lemna turionifera* 5511) increases starch production by promoting light and nitrogen utilization [15]. Our investigation into *PSAT*’s transcriptomic response to Cd stress revealed the downregulation of most genes in glycolysis and the TCA cycle, potentially resulting from biochemical reactions triggered by stress-induced injury. Key enzymes involved in Glu synthesis, including *PSAT*, GS, and GOGAT, exhibited significant upregulation, contrasting with the downregulation observed for GAD (Figure 4). These findings imply that *PSAT* orchestrates Glu synthesis and metabolism under Cd stress, exerting regulatory influence in this context. The metabolomics results showed that *PSAT* had the highest content of Glu under Cd stress (Figure 3b), which is consistent with the transcriptomic results.

Previous studies have indicated that Glu can form complexes with Cd, enhancing the Cd enrichment ability of rice roots [5]. We quantified Cd content in the treatment solution of *PSAT* and WT, along with Cd accumulation in duckweed. The results revealed that *PSAT* overexpression bolstered Cd absorption in duckweed roots, with no marked distinction in leaf Cd content (Figure 6a,c). NMT experiments showed that Cd absorption at the *PSAT* root was significantly stronger than in WT, while no variation was evident in the leaves (Figure 6b). The transport of Cd may be influenced by the activity of transporters present in roots and the phloem membrane. The elevated Glu content emerges as a plausible explanation for *PSAT*’s enhanced Cd absorption capability in duckweed.

More than a metabolite molecule, Glu plays a role as signaling molecule, and is involved in plant seed germination, establishment, growth, flower, and senescence, as well as response to stress [4]. What is noteworthy is that the Glu concentration is important. Hence, the improvement of Glu metabolic enzymes is important for plants during stress to maintain the balance of Glu concentration, which is consistent with our results.

### 3.3. Overexpression of PSAT Promoted the Antioxidant System Capacity under Cd Stress

Cd stress triggers the generation of numerous reactive oxygen species (ROS) in plants, resulting in the inhibition of enzyme activity and the disruption of cell membrane integrity through interactions with lipids, proteins, and nucleic acids [26]. Among antioxidant enzyme activities, non-enzymatic antioxidants play a crucial role, with glutathione (GSH) being the most prominent. Maintaining a high GSH/GSSG ratio is essential for responding to oxidative stress in plants [27,28,29]. In line with our hypothesis, the Evans blue staining experiment revealed that overexpression of *PSAT* significantly reduced cell membrane damage compared to WT (Figure 2d). Measurement of CAT and SOD activity or content in *PSAT* under Cd stress indicated a significant increase in all parameters (Figure 3a). Under Cd stress, WT exhibited a burst of ROS, resulting in a series of damages. Therefore, Evans Blue staining in the leaves showed a deeper blue color in WT under Cd stress, suggesting greater damage to the cell membrane in WT with Cd. There was also a decline in the activity of protective enzymes in WT during Cd stress. In contrast, *PSAT* showed elevated levels of GSH, effectively preventing further escalation of ROS. The ROS level increased in WT, and the levels of protective enzymes decreased, resulting in membrane damage. This substantiates our speculation that *PSAT* mitigates Cd stress-induced harm by strengthening the antioxidant system in duckweed. Metabolomics analysis highlighted higher GSH and GSSG levels in *PSAT* under non-Cd stress conditions (Figure 3b). We believe that the elevated GSH and GSSG levels in *PSAT* establish a foundation for non-enzymatic antioxidant defense against Cd stress.

## 4. Materials and Methods

### 4.1. Duckweed Culture

Duckweed (*Lemna turionifera* 5511) was cultured in a liquid medium following previously established protocols [30,31]. The medium’s pH was maintained within the range of 5.8–6.0, and it was sterilized at 121 °C for 20 min before use. Duckweed growth conditions were maintained at 23 °C during the day and night, with 16 h of light at an intensity of 95 μmol m^−2^·s^−1^ and 8 h of darkness per day.

### 4.2. Transgenic and Plant Tissue Culture

The transformation of the *AtPSAT* gene was carried out through Agrobacterium-mediated transformation on duckweed callus. In brief, fully expanded leaves cultured for 10 to 15 days were selected as callus-inducing explants. The induction medium consisted of B5 medium with 1.5% sucrose, 15 mg/L dicamba, 3.5 mg/L 2,4-dichlorophenoxyacetic acid (2,4-D), and 1 mg/L 6-benzyladenosine (BA). After 2–3 weeks of induction, the callus was transferred to B5 culture medium containing 1.5% sucrose, 10 mg/L 4-chlorophenoxyacetic acid (CPA), and 2 mg/L 6-(γ, γ-dimethylacrylamide)-purine (2ip) for subculture [31]. The callus was refreshed with fresh medium every 14 days. Callus transformation was then performed following the method described by [32]. After co-cultivation without light for 3 days, the callus was washed with sterile water containing 300 mg/L cefotaxime and transferred to a selective maintenance medium containing 30 mg/L hygromycin. Finally, the callus was transferred to a selective regeneration medium, which included B5 medium with 1.5% sucrose, 1 mM L-Ser, and 1 mg/L hygromycin. Each regenerated leaf was transferred to a liquid medium containing 1 mg/L hygromycin until young leaves grew from the callus, and it was cultured into a single transformation line for DNA molecular identification.

### 4.3. Binary Vector Construction and Agrobacterium Transformation

The *PSAT* gene was obtained from *Arabidopsis thaliana* through reverse transcription. pCAMBIA 1301-*AtPSAT* was created by inserting the target gene *AtPSAT* between the CaMV 35s promoter and NOS terminator using PCR and restriction enzyme digestion with *Nco*Ⅰ and *Bst*EⅡ at 37 °C (Figure 1a). pCAMBIA 1301-*AtPSAT* was transferred into *Agrobacterium tumefaciens* using the fast freeze–thaw method [33]. DNA samples from duckweed were extracted using a Tiangen kit (DNA kit, TIANGEN, Beijing, China). The PCR experiment was carried out in a 50 μL volume, including 2 μL of the template, 10 μL of Premix Taq, 1 μL of the forward primer, 1 μL of the reverse primer, and 6 μL of ddH2O, followed by 30 cycles at 98 °C for 10 s, 55 °C for 30 s, and 72 °C for 1 min. The primers used were as follows:

F 5′-ATGGCGGCTACGACGAACT-3′; R 5′-CTAAGCATGCTTAGCCTGG-3′.

### 4.4. Determination of Chlorophyll Content

To determine the chlorophyll content, both WT and *PSAT* duckweeds were subjected to 50 μM Cd treatment for 48 h. A total of 200 mg of duckweed was collected and immersed in 25 mL of 95% alcohol for 24 h. Chlorophyll content was measured at 663 nm and 645 nm using a spectrophotometer. Chlorophyll a (Chla), chlorophyll b (Chlb), and total chlorophyll (Chl a + b) were calculated as follows [34]:

Chla (mg/g) = {(12.7A663 − 2.69A645) ∗ 25 mL}/0.2 g

Chlb (mg/g) = {(22.9A645 − 4.68A663) ∗ 25 mL}/0.2 g

Chl (a + b) (mg/g) = {(18.02A663 − 20.21A645) ∗ 25 mL}/0.2 g [34].

### 4.5. Determination of Cd Content in Medium

WT and *PSAT* duckweed were exposed to 50 μM Cd with a final volume of 50 mL for 24 h. The solution was subsequently acidified with 3% nitric acid, and the Cd content was measured using an inductively coupled plasma emission spectrometer (ICP). Standard curve concentrations ranged from 0, 0.001 and 0.01 to 0.1 mg/L.

### 4.6. Cd^2+^ Flux Determination

The non-invasive micro-test technique (NMT) was used to obtain ion and molecular signals through flow rate microsensors. It calculated the concentration and flow rate of dissociated molecules based on the Nernst equation and Fick’s first diffusion law. This technique allows for the detection of very subtle signals, with flow rates reaching 10–12 mol·cm^−2^. Both WT and *PSAT* were exposed to 50 μM Cd for 30 min. NMT was employed to measure the Cd^2+^ flux in root tips and mesophyll cells using test solutions consisting of 50 μM Cd, 0.1 mM CaCl_2_, 0.1 mM KCl, and 0.3 mM MES at pH 5.8.

### 4.7. Evans Blue Dyeing

After treating both WT and *PSAT* duckweeds with 50 μM Cd for 48 h, the plant tissues were subjected to staining with 0.25% Evans blue for 24 h. Subsequently, decolorization was carried out using 1 mL of decolorization solution (Ethanol: Acetic acid = 3:1) in a 1.5 mL centrifuge tube for 6 h. The decolorization solution was changed every 2 h until complete decolorization was achieved. The samples were then rinsed with PBS (phosphate-buffered saline) 3–5 times, and the roots and leaves were separated. Finally, the stained tissues were observed under a microscope (Leica DFC450C, DM5000, Wetzlar, HE, Germany).

### 4.8. Antioxidant Activity

The determination of superoxide dismutase (SOD) and catalase (CAT) activities was performed using double-antibody sandwich enzyme-linked immunosorbent assay (ELISA) kits from Enzyme-linked Biotechnology Co., Shanghai, China. Purified plant SOD/POD/CAT antibodies were coated on microporous plates to create the solid-phase first antibody. Plant samples from different groups were ground with 0.1 g of fresh weight in liquid nitrogen and then centrifuged with 1 mL of PBS to create a suspension. The supernatant was diluted 5 times before being used for sampling. The samples were incubated at 37 °C for 30 min and then washed with washing solution 20 times for 5 min each. Next, horseradish peroxidase (HRP)-labeled detection antibodies were added to form an antibody–antigen–enzyme conjugate antibody complex, which was incubated for 30 min and then washed. Finally, the substrate 3,3′,5,5′-Tetramethylbenzidine (TMB) was added for color development, and the absorbance (OD) was measured at a wavelength of 450 nm using an enzyme-labeled instrument.

### 4.9. Flow Cytometric Analysis of Cd Content in Roots

The duckweed was soaked in 95% ethanol for 15 min; then, the roots and leaves were separated. Initially, 95% ethanol was used to preserve the treated duckweed for 10 min. The fronds and roots of the duckweed were separated, and the roots were designated for subsequent treatments. Subsequently, they were incubated in the dark at 37 °C for 60 min with a mixture of 1% cellulase and 1% pectinase to generate protoplasts, which were then rinsed three times using DPBS (Dulbecco’s phosphate-buffered saline). Afterwards, Cd levels were assessed using 30 µL of Leadmium^TM^ Green AM dye at 37 °C for 60 min. Following staining, the cells were washed three times with DPBS and passed through a 400-mesh cell filter. Intracellular Cd content was quantitatively determined using flow cytometry (Merck Millipore, FlowSight^®^ Imaging Flow Cytometry, Darmstadt, HE, Germany).

### 4.10. RNA Sequencing and Analysis

Following 24 h of Cd treatment, the RNA from both the WT and *PSAT* groups underwent sequencing and analysis at Novogene in Chaoyang, Beijing, China. Total RNA was collected using the QIAGEN Total RNAprep Pure Plant Kit (Beijing, China). The quality of the RNA was assessed using an Agilent 2100 Bioanalyzer (Santa Clara, CA, USA). Subsequently, the library was constructed and examined using the Illumina NovaSeq 6000 sequencing system (San Diego, CA, USA). The samples were subjected to gene expression analysis using transcriptomic methods at Novogene in Chaoyang, Beijing [30]. Gene functional annotation was performed using the following databases: gene ontology (GO), the KEGG Ortholog database (KO), and the manually annotated and reviewed protein sequence database (Swiss-PROT) [35]. Expression levels were analyzed following Dewey and Bo.

### 4.11. Untargeted Metabolom Analysis

Tissues (100 mg) were individually ground with liquid nitrogen, and the homogenate was resuspended with prechilled 80% methanol using a well vortex. The samples were incubated on ice for 5 min and then centrifuged at 15,000× *g* and 4 °C for 20 min. Some of the supernatant was diluted to a final concentration containing 53% methanol using LC-MS grade water. The samples were subsequently transferred to a fresh Eppendorf tube and then centrifuged at 15,000× *g* and 4 °C for 20 min. Finally, the supernatant was injected into the LC-MS/MS system analysis.

UHPLC-MS/MS analyses were performed using a Vanquish UHPLC system (Thermo Fisher, Waltham, MA, USA) coupled with an Orbitrap Q ExactiveTMHF mass spectrometer (Thermo Fisher, Waltham, MA, USA). Samples were injected onto a Hypersil Gold column (100 × 2.1 mm, 1.9 μm) using a 17 min linear gradient at a flow rate of 0.2 mL/min. The eluents for the positive polarity mode were eluent A (0.1% formic acid in water) and eluent B (methanol). The eluents for the negative polarity mode were eluent A (5 mM ammonium acetate, pH 9.0) and eluent B (methanol). The solvent gradient was set as follows: 2% B, 1.5 min; 2–85% B, 3 min; 85–100% B, 10 min; 2–100% B, 10.1 min; 2% B, 12 min. A Q ExactiveTM HF mass spectrometer was operated in positive/negative polarity mode with a spray voltage of 3.5 kV, a capillary temperature of 320 °C, a sheath gas flow rate of 35 psi, an aux gas flow rate of 10 L/min, an S-lens RF level of 60, and an Aux gas heater temperature of 350 °C.

### 4.12. Statistical Analysis

All experiments were repeated in at least triplicate, with more than 20 leaves per group of parallel experiments. Experimental data were organized using Microsoft Excel 2010 and plotted using GraphPad Prism 8 and Adobe Illustrator 2020. Variables were subjected to independent sample tests and one-way ANOVA in SPSS software (IBM SPSS Statistics, Version 26). Significant differences are indicated by asterisks (* *p* < 0.05, ** *p* < 0.01).

## 5. Conclusions

In this study, *PSAT* was overexpressed in duckweed, resulting in improved Cd tolerance and Cd accumulation capabilities. *PSAT* transgenic duckweed could be applied for future Cd remediation efforts. With increased Glu content, *PSAT* duckweed mitigated phenotypic damage under Cd stress, upregulated the expression of photosynthetic system-related genes, and raised chlorophyll levels. Furthermore, it bolstered both enzyme and non-enzyme antioxidant systems to mitigate oxidative damage during Cd exposure. *PSAT* promoted glutamate metabolism and Cd enrichment. This study provides a theoretical foundation for harnessing duckweed as a potent candidate for environmental remediation, given its impressive capacity for Cd accumulation and tolerance.

## Figures and Tables

**Table 1:** Changes in gene expression levels related to photosynthesis and antenna proteins.

Description	Gene_id	WT Readcount	WT_Cd_ Readcount	*PSAT*_ Readcount	*PSAT*_Cd_ Readcount
photosystem II oxygen-evolving enhancer protein 1 (psbO)	Cluster-10487.13290	63,495.69	6548.87	53,162.82	9763.02
photosystem II oxygen-evolving enhancer protein 2 (psbP)	Cluster-10487.13349	71,702.28	4429.11	56,594.10	6301.73
photosystem II oxygen-evolving enhancer protein 3 (psbQ)	Cluster-10487.13567	34,069.38	4052.00	26,869.70	6230.79
photosystem II 22kDa protein (psbS)	Cluster-10487.12967	47,665.68	8263.34	40,112.19	16,756.60
photosystem II Psb27 protein (psb27)	Cluster-10487.12473	8163.18	660.61	6610.15	1066.84
photosystem I P700 chlorophyll a apoprotein A2 (psaB)	Cluster-10487.11012	327.32	91.44	379.35	192.81
photosystem I subunit II (psaD)	Cluster-10487.13383	24,208.90	1333.68	21,347.69	2571.46
photosystem I subunit III (psaF)	Cluster-10487.13871	40,494.00	1441.59	34,514.82	2883.82
photosystem I subunit V (psaG)	Cluster-10487.14019	20,411.66	1174.12	16,809.05	2253.86
photosystem I subunit X (psaK)	Cluster-10487.13026	58,235.24	3143.84	52,249.92	4969.48
photosystem I subunit XI (psaL)	Cluster-10487.12136	25,777.67	153.51	1351.28	396.65
photosystem I subunit PsaN (psaN)	Cluster-10487.13217	11,538.31	282.10	9799.61	523.40
photosystem I subunit PsaO (psaO)	Cluster-10487.13357	50,900.95	2350.57	42,577.72	4372.08
PlastocyaninPetE	Cluster-10487.11211	870.30	132.36	677.34	334.52
Ferredoxin—NADP+ reductase (PetH)	Cluster-10487.13830	25,777.67	3549.37	21,423.32	5857.56
F-type H^+^−ransporting ATPase subunit alpha (alpha)	Cluster-10487.17966	202.99	42.64	250.32	149.68
F-type H^+^−transporting ATPase subunit gamma	Cluster-10487.13124	34,408.50	5090.39	28,765.28	7295.34
F-type H^+^−transporting ATPase subunit delta	Cluster-10487.13237	11,239.41	1307.13	11,155.56	1891.45
F-type H^+^−transporting ATPase subunit b	Cluster-10487.12864	9853.74	895.69	9421.21	1537.34

## Data Availability

The original contributions presented in the study are included in the article; further inquiries can be directed to the corresponding author.

## Supplementary Materials

The following supporting information can be downloaded at: https://www.mdpi.com/article/10.3390/plants13050627/s1, Figure S1:The relative contents of (5-L-Glutamyl)-L-amino acid and 5-Oxoproline in WT /PSAT and WT/PSAT were treated with Cd; Table S1: Changes in gene expression levels relates to the TCA cycle glycolysis, glutamic acid, and serine metabolic pathway *(PSAT* vs. WT vs. *PSAT*-Cd vs. WT-Cd); Table S2: Glu and GABA relative content in PSAT and WT with or without Cd; Table S3: Changes of peroxisome gene expression under Cd stress.
