# Supplementary material for: Overexpression of Phosphoserine Aminotransferase (PSAT)-Enhanced Cadmium Resistance and Accumulation in Duckweed (Lemna turionifera 5511)
Source: Plants (Basel). 2024 Feb 25;13(5):627. doi: 10.3390/plants13050627 (PMC10934196; doi:10.3390/plants13050627)
Supplement: Supplementary file 1 [file plants-13-00627-s001.zip › plants-2825935-supplementary.pdf]

Table S1 Changes in gene expression levels relates to the TCA cycle Glycolysis, Glutamic acid and serine metabolic pathway. (PSAT vs WT vs PSAT-Cd vs WT-Cd)

	NR n	gene_id	PSAT_re	WT_reac	PSAT_Cd_r	WT_Cd_readco
GOGOAT	ferredoxin-dependent glutamate synthase, chloroplastic isoform X2 [Phoenix dactylifera]	Cluster-10487.10799	1791.98	2057.36	394.93	590.99
		Cluster-10487.13202	17336.15	18761.30	2068.21	1624.72
		Cluster-10487.13325	7740.66	9365.12	11895.84	9371.78
		Cluster-10487.14376	120.61	134.31	2074.63	937.35
GDH	Os03g0794500 [Oryza sativa Japonica Group]	Cluster-10487.11995	2439.15	2830.49	3348.21	5356.46
GAD	glutamate decarboxylase 1 [Sorghum bicolor]	Cluster-10487.11944	4027.15	6228.14	47687.37	64436.53
PSAT1	Phosphoserine aminotransferase 2, chloroplastic [Ananas comosus]	Cluster-10487.5453	155.50	169.28	82.57	144.74
GS	glutamine synthetase 2 [Dunaliella viridis]	Cluster-4255.0	4.32	0.00	4.77	0.00
		Cluster-10487.13188	7308.27	8537.42	912.68	651.78
		Cluster-10487.2592	51.08	0.00	10.47	0.00
		Cluster-10487.12791	9190.29	12581.87	5371.51	3123.81
		Cluster-10487.15584	3111.01	3739.58	3693.76	1927.86
		Cluster-10487.12443	10769.28	14015.19	2376.44	1560.68
		Cluster-10487.12691	7451.58	4850.70	3407.23	2382.54
PSPH	PREDICTED: BTB/POZ domain-containing protein At1g55760-like [Musa acuminata subsp. malaccensis]	Cluster-10487.7071	432.47	390.13	1706.09	1748.33
PDGH	D-3-phosphoglycerate dehydrogenase 1, chloroplastic [Cinnamomum	Cluster-10487.10771	794.29	925.40	5063.33	5230.99
		Cluster-10487.12066	488.58	588.69	314.72	313.14

	micranthum f. kanehirae]	Cluster-10487.14483	1872.09	1976.45	1112.29	983.79
		Cluster-11945.0	0.00	1.46	0.00	0.99
		Cluster-12045.0	5.14	0.00	0.34	0.00
		Cluster-3581.0	0.00	0.00	0.68	0.00
		Cluster-3767.0	6.62	0.00	1.70	0.00
		Cluster-7219.1	1.28	0.00	0.00	1.38
PK	pyruvate kinase, cytosolic isozyme-like [Durio zibethinus]	Cluster-10487.13167	1188.12	1423.55	3401.08	4664.00
		Cluster-10487.14362	3586.78	3878.10	7030.98	8493.10
		Cluster-10487.2961	35.98	0.00	12.09	0.00
HK	hexokinase [Salvia splendens]	Cluster-10487.15212	1501.14	1861.25	1350.71	1782.59
GPI	glucose-6-phosphate isomerase 1, chloroplastic [Phoenix dactylifera]	Cluster-10487.14550	720.53	745.31	489.16	477.25
		Cluster-10487.24281	0.00	0.00	0.55	0.00
		Cluster-10487.9551	1215.84	1433.37	1809.12	2080.67
		Cluster-12711.0	0.69	5.44	0.28	3.73
		Cluster-5686.0	0.00	0.00	0.83	0.00
PFK	ATP-dependent 6-phosphofructokinase 3 isoform X1 [Elaeis guineensis]	Cluster-10487.8598	290.81	287.64	476.05	648.04
		Cluster-10487.10246	515.97	378.90	260.37	420.79
		Cluster-10487.12008	3546.23	3855.19	1743.66	2614.43
		Cluster-10487.14434	6433.37	6805.32	9679.87	14685.10
ALD	PREDICTED: fructose-bisphosphate aldolase 6, cytosolic [Eucalyptus grandis]	Cluster-10487.14165	7605.96	8385.39	4926.97	8494.13
		Cluster-10487.13219	9331.18	10264.98	10294.36	13547.15
GAPDH	glyceraldehyde-3-phosphate dehydrogenase [Ananas comosus]	Cluster-10487.13988	25179.82	29311.09	21799.99	30715.26
		Cluster-10487.10314	1111.28	1112.82	700.51	489.78
		Cluster-10487.10780	6712.20	9311.48	3239.83	2157.29

		Cluster-10487.12307	8131.55	10140.51	973.17	593.45
		Cluster-10487.12391	8046.07	11294.53	1572.67	1099.05
		Cluster-10487.13288	28896.45	42266.05	6608.02	5189.38
		Cluster-10487.13988	25179.82	29311.09	21799.99	30715.26
PGK	Phosphoglycerate kinase [Macleaya cordata]	Cluster-10487.13010	5370.79	5878.97	6015.94	8161.46
PGAM	2,3-bisphosphoglycerate-independent phosphoglycerate mutase [Elaeis guineensis]	Cluster-10487.9542	44.24	118.17	83.19	225.57
		Cluster-10487.13460	3961.64	3952.75	7999.80	11120.54
ENO	PREDICTED: cytosolic enolase 3 isoform X1 [Nelumbo nucifera]	Cluster-10487.17179	462.87	382.19	339.11	454.09
PDC	dihydrolipoyllysine-residue acetyltransferase component 1 of pyruvate dehydrogenase complex, mitochondrial [Asparagus officinalis]	Cluster-10487.13033	1215.22	1291.26	3874.51	5658.01
		Cluster-10487.15251	484.82	450.88	325.47	458.42
citrate synthase	uncharacterized protein A4U43_C08F34760 [Asparagus officinalis]	Cluster-10487.15095	1048.94	1262.36	3250.67	4385.44
		Cluster-10487.17713	669.47	651.26	2289.36	3181.60
ACD	aconitate hydratase 1 [Papaver somniferum]	Cluster-10487.10751	815.49	859.05	1830.52	2402.57
		Cluster-10487.14461	526.10	486.46	583.15	800.94
		Cluster-10487.2884	71.66	0.00	9.22	0.00
IDH	Isocitrate dehydrogenase [NAD]	Cluster-10487.11810	1181.31	1657.77	2656.21	3422.07

	catalytic subunit 5, mitochondrial	Cluster-10487.14298	2154.51	2383.88	6727.59	8569.47
	[Dendrobium catenatum]	Cluster-10487.15573	1774.59	2238.38	4327.61	5729.43
2OG-DH	PREDICTED: dihydrolipoyllysine-residue succinyltransferase component of 2-oxoglutarate dehydrogenase complex 2, mitochondrial-like [Nelumbo nucifera]	Cluster-10487.11240	2404.13	2481.16	3160.27	4231.67
SDH	succinate dehydrogenase [ubiquinone] flavoprotein subunit, mitochondrial isoform X1 [Elaeis guineensis]	Cluster-10487.12588	429.19	416.89	369.27	478.58
		Cluster-10487.14291	4492.31	4497.92	1720.87	1357.18
		Cluster-10487.15233	1176.86	1218.00	2336.70	2821.08
		Cluster-10487.15330	2082.85	2267.11	4891.99	6826.58
FUM	PREDICTED: fumarate hydratase 1, mitochondrial [Musa acuminata subsp. malaccensis]	Cluster-10487.13515	1099.99	969.87	829.46	1163.78
MDH	malate dehydrogenase, glyoxysomal [Cinnamomum micranthum f. kanehirae]	Cluster-10487.13861	2526.79	3032.15	2512.61	3222.38
		Cluster-10487.13163	7970.34	9601.99	4252.99	3367.19
		Cluster-10487.7802	474.30	605.80	192.69	26.14
CS	hypothetical protein CUMW_136650 [Citrus unshiu]	Cluster-10487.17713	669.47	651.26	2289.36	3181.60
		Cluster-10487.15095	1048.94	1262.36	3250.67	4385.44

Table S2 Glu and GABA relative content in PSAT and WT with or without Cd

	Average Glu Content	Standard deviation of Glu	Relative Glu Content	Average GABA Content	Standard deviation of GABA	Relative GABA Content
WT	83.8858	±6.0168	1.0000	0.3748	±0.0875	1.0000
PSAT	117.5645	±7.6984	1.4015	0.3053	±0.0363	0.8145
WT-Cd	113.3219	±6.2450	1.3509	0.4964	±0.1375	1.3244
PSAT-Cd	149.3919	±17.4213	1.7809	0.4374	±0.0625	1.1671

Table S3 Changes of peroxisome gene expression under Cd stress

	NR Description	gene_id	PSAT_read count	WT_read count	PSAT_Cd_readcount	WT_Cd_readcount
PMP34	hypothetical protein POPTR_002G000200 [Populus trichocarpa]	Cluster-10487.18022	379.9958633	308.821903	831.4500398	584.8055564
MPV17	uncharacterized protein LOC109708813 [Ananas comosus]	Cluster-10487.9507	887.204278	910.6209694	1868.223621	1259.128221

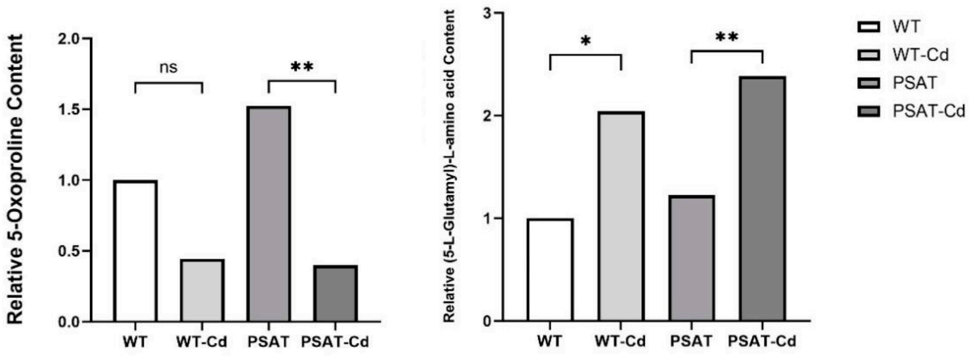

Fig. S1 The relative contents of (5-L-Glutamyl)-L-amino acid and 5-Oxoproline in WT /PSAT and WT/PSAT were treated with Cd. (* $p < 0.05$, ** $p < 0.01$)
